# Medical Opinion and Movement

**Published:** 1907-01-19

**Authors:** 


### Medical Opinion and Movement.

Rontgen Ray operators are still sadly in need of some standard of measurement for $x$-ray effects. This is especially necessary for the comparison of the effects upon the tissues, so that different operators may be able to form a definite idea of the variations in each other's work, or in their own work under different conditions. A committee has been formed by the Röntgen Society to investigate the matter, and, if possible, to devise some method of standardisation. In a recent address on the subject the President of the Society, Mr. C. V. Boys, F.R.S., has emphasised the many difficulties of the task to which they are committed. A standard of absolute units expressed in the C.G.S. system would be the ideal one, but this appears altogether beyond our reach at present. There is no known relation between the total energy of the rays or their heat of absorption, and their therapeutic, chemical, or photographic effects, and these effects vary for the different wave lengths, so that it is not possible to express the measure of the effects in terms of heat units. In order to provide an absolute standard it will therefore be necessary to determine exactly what physical property or properties are responsible for the different phenomena. In the meantime we shall have to content ourselves with some arbitrary standard. This, however, is a problem of sufficient difficulty. Vacuum tubes are found to differ in respect of many qualities, such as the "hardness" or "softness" of the $x$-rays they emit, and one effect as observed on the skin seems to have no relation to other effects measured by various instruments. The hope of a solution to the problem appears to lie in the discovery of some definite relation between the effect of the rays on the tissues and some special physical manifestation.

Hypnotism cannot be said to have yet received formal and authoritative recognition as a therapeutic measure by the profession. It has been found difficult to differentiate the purely scientific phenomena of the hypnotic sleep from the charlatanism and trickery that have grown up around the subject. Misconceptions and prejudices have undoubtedly done much to prevent a proper estimate of the worth of hypnotism. In spite of many able exponents among members of the medical profession both here and on the Continent, there is still a large proportion of medical men who look askance at its practice. In New Zealand the subject of hypnotic treatment has been recently brought before the profession at the Medical Congress by Dr. P. Clennell Fenwick, surgeon to the Wanganui Hospital. He has only used hypnotic suggestion in his practice during the past year, but in that short time he claims to have achieved some notable results. Cases of asthma, constipation, chorea, psoriasis, as well as various neuroses, have yielded to the charm of hypnotic suggestion. In addition to these interesting curative phenomena, Dr. Fenwick was able to produce a photograph of a hand showing two blisters raised by suggestion during the hypnotic sleep. This experiment was performed by Dr. Otto G. Wettestrand. The blisters appeared seven hours after the suggestion, and it is stated that four Swedish physicians were present during the whole time. This ability to produce a blister by hypnotic suggestion has been claimed by a few operators from time to time, but the evidence has not been considered altogether free from criticism. Indeed, such an experienced operator and authority as Dr. Bramwell states that he has never been able to produce this phenomenon, and throws considerable doubt upon the possibility of its achievement. If mental suggestion can actually produce such a lesion of the skin, there seems no reason why such a power should not be utilised to remove existing disease.

At the recent opening of a new wing to the National Maternity Hospital in Dublin, his Grace the Archbishop of Dublin took the opportunity to comment on the inequitable distribution of the parliamentary grant to the Dublin hospitals. It is unfortunately one of those Irish grievances which, in spite of repeated commissions of inquiry, and in spite also of definite recommendations of reform, remain unredressed. As stated by his Grace the Archbishop, the facts of the case are that of the £15,850 of public money voted by Parliament every year as a State subvention to the hospitals of the city, every penny is bestowed upon hospitals exclusively or predominantly Protestant, the claims of the Catholic hospitals being entirely ignored. It is further alleged that such is the sectarian spirit pervading the controlling hospital authorities that the position of master to the Rotunda Hospital is a post to which no Catholic doctor would have the slightest chance of being appointed. The only proper basis for distribution of public money to the hospitals is one calculated in accordance with the amount and quality of work carried out by them, provided they administer to any of the community in need of their assistance, and independently of religious persuasion. The reform of a system of distribution already in existence for a considerable period doubtless presents many questions of difficulty. To cut down supplies to certain institutions, after they have depended upon these for so many years, would inflict upon such institutions more severe hardships than if they had never been recipients of the grants, and might in many cases seriously curtail the good work they are doing. The only alternative would be to increase the amount of the subvention to such a sum as would allow of a proportionate distribution to those hospitals not now included in the grant. Any additional grant must however depend upon the relative average efficiency of every institution included in the list of recipients. No grants ought to be made except they are based upon the report of competent inspectors who have visited each hospital and are able to judge the quality of the work and management.

